# A human mission to Mars: Predicting the bone mineral density loss of astronauts

**DOI:** 10.1371/journal.pone.0226434

**Published:** 2020-01-22

**Authors:** Eneko Axpe, Doreen Chan, Metadel F. Abegaz, Ann-Sofie Schreurs, Joshua S. Alwood, Ruth K. Globus, Eric A. Appel

**Affiliations:** 1 Space Biosciences Division, NASA-Ames Research Center, California, United States of America; 2 Department of Materials Science & Engineering, Stanford University, Stanford, California, United States of America; 3 Department of Chemistry, Stanford University, Stanford, California, United States of America; 4 Department of Bioengineering, Stanford University, Stanford, California, United States of America; Charles P. Darby Children's Research Institute, UNITED STATES

## Abstract

A round-trip human mission to Mars is anticipated to last roughly three years. Spaceflight conditions are known to cause loss of bone mineral density (BMD) in astronauts, increasing bone fracture risk. There is an urgent need to understand BMD progression as a function of spaceflight time to minimize associated health implications and ensure mission success. Here we introduce a nonlinear mathematical model of BMD loss for candidate human missions to Mars: (i) Opposition class trajectory (400–600 days), and (ii) Conjunction class trajectory (1000–1200 days). Using femoral neck BMD data (N = 69) from astronauts after 132-day and 228-day spaceflight and the World Health Organization’s fracture risk recommendation, we predicted post-mission risk and associated osteopathology. Our model predicts 62% opposition class astronauts and 100% conjunction class astronauts will develop osteopenia, with 33% being at risk for osteoporosis. This model can help in implementing countermeasure strategies and inform space agencies’ choice of crew candidates.

## Introduction

A fracture occurs when a certain applied load exceeds the ultimate strength of the bone. A measurable, critical component for the bone strength is the areal bone mineral density (BMD) [1]. BMD loss is an established fracture risk factor due to bone weakening as measured in large epidemiological studies correlating BMD with the incidence of fragility fractures [2–4]. Current standards to monitor and maintain bone health in both NASA and ISS crewmembers are based in BMD measurements by dual-energy x-ray absorptiometry (DXA) [5]. It is well known that long-duration spaceflights induce BMD loss in weight-bearing bones although there is considerable individual variability [6–12]. BMD loss as a function of spaceflight duration remains poorly understood, with an average rate of loss typically between 1–1.5%/month for weight bearing bones [13,14]. These calculations are based in DXA measurements before and after missions usually of 4–6 months in length.

During the >54 million kilometers traversed during a mission to Mars, astronauts will be exposed to the longest microgravity and cosmic radiation conditions in the history of spaceflight. Thus, defining potential health implications of such exposure is necessary before proceeding with interplanetary explorations [15]. In fact, the International Space Exploration Coordination Group (ISECG), comprised of 14 space agencies, aims to advance a long-range human space exploration strategy. According to the latest edition of the ISECG Global Exploration Roadmap [16], released in January 2018, a mutual driving objective between agencies is to enable human presence on Mars. NASA’s first human mission to the vicinity of Mars is expected for the mid-2030s, and a human landing on the Martian surface is anticipated for the 2040s.

A predictive mathematical model for BMD loss during long-duration spaceflights could help space agencies make decisions about mission duration and crew selection to minimize fracture risk. To our knowledge, each and every prior work assume a linear decrease of the BMD in astronauts to make predictions [13], which is not valid for long duration spaceflights. Specifically, BMD loss in the femoral neck has been modeled as a linear decrease: (1.06 ± 0.63)% BMD loss per month [17,18]. However, a linear decrease is not realistic for very long duration, interplanetary missions, as it predicts nonphysical negative BMD values for longer time spans. In contrast, BMD recovery on Earth in the bones of various astronauts after spaceflights is well described by an exponential function [19]. Furthermore, there is terrestrial evidence that the loss of bone matrix and bone mineral due to conditions that result prolonged bed rest (*e*.*g*. disuse medical conditions and/or spinal cord injury) eventually plateaus at 69.0% initial BMD, after a period of significant and progressive decline [18,20,21]. Thus, a non-linear, exponential decline of BMD in weight-bearing bones is a reasonable approach to model progressive bone loss in long-duration space missions.

In this paper, we introduce for the first time a predictive mathematical model for the BMD loss defined by an exponential decrease in load bearing bones of the astronauts. By using this model, we predict BMD loss in the femoral neck for two potential missions to Mars.

## Methods

### Mathematical formulation

There is terrestrial evidence that bone density is likely to plateau after a long period of loss in weight-bearing bones [18,20,21]. Further, there are no supporting data from astronauts available for time-frames relevant to interplanetary travel (all the candidate missions are in the order of years given current rocket capabilities). However, spinal cord injury provides an example where a plateau in BMD decrements occurs 1.5–2.5 years post-injury, which is similar to the total duration of a potential human mission to Mars. Furthermore, previous studies demonstrated that the alterations that occur following spinal cord injury and exposure to microgravity are remarkably similar [22]. Therefore, we formulate that the change of areal BMD in weight-bearing bones at time t, BMD(t), during spaceflights as a one-phase exponential decay:

$$BMD\%(t)=(C-P)e^{-\lambda t}+P$$

BMD change is here expressed as percentage [%], *λ* corresponds to the BMD decay rate, and C to the areal bone mineral density right before launch:

C=BMD%(t=0)=0%

P corresponds to the plateau. The maximum total BMD loss has been previously estimated to be 69.0% relative to the astronauts’ pre-flight BMD [18], and obtained by combining data from different large studies in humans [23,24]. Therefore, assuming that the BMD loss will eventually plateau at this maximum value:

$$BMD\%(t)=(-69.0\%)e^{-\lambda t}+69.0\%$$

We performed a nonlinear regression of the available data for BMD loss in the femoral neck. In order to obtain the decay rate (*λ*) of the BMD of the femoral neck in our model, we defined the plateau as *P* = 69.0% BMD loss compared to prelaunch. Femoral neck was chosen between other weight-bearing bones because it is the recommended by the World Health Organization (WHO) [25] to determine the fragility fracture risk associated T-score, as well as to diagnose osteoporosis in clinical practice [18]. Table 1 includes all the data currently available in the literature (69 values in total, some expressed as mean ± SD, as indicated).

**Table 1:** Percent loss in bone mineral density at femoral neck in astronauts (N = 69) after 132- to 228-day spaceflights.

**Time of spaceflight, t (days)**	**Bone mineral density loss in femoral neck (%)**	**Number of astronauts in the study (n)**	**Reference**
11	0	69	[32,33]
132	-1.3	1	[32]
132	-6.0	1	[32]
145	-4.5	1	[32]
150 ± 30	-9.4 ± 6.4	16	[33]
169	-3.5	1	[32]
169	-3.1	1	[32]
176	-11.4	1	[32]
176	-5.3	1	[32]
181 ± 47	-6.8 ± 1.1	46	[19]

Once we calculated BMD loss by using the previous equation, we calculated the T-score for different ethnicities and sexes by using normative data from the *National Health and Nutrition Examination Survey* (NHANES) reference database, exactly as recommended by the World Health Organization. T-score is defined as the number of standard deviations above or below the mean for Caucasian women, aged 20–29 years (*<BMD*_*ref*_ *>* = 0.858, SD = 0.120):

$$T_{score}=(<{BMD}_{post-Mission}>-0.858)/0.120$$

In the previous formula, <*BMD*_*post*−*Mission*_> was calculated for each group *i*, as:

$$<{BMD}_{i,post-mission}>=\left(<{BMD}_{i,pre-mission}>-\frac{100-BMD\%(t)}{100}\right)$$

Here, <*BMD*_*i*,*pre*−*mission*_> were obtained as the mean values of each group (male/female, non-Hispanic, white/non-Hispanic, black, Mexican-American and 30–39 years, 40–49 years) from the NHANES reference database. The BMD%(t) corresponds to the BMD loss obtained in this study. All the obtained data can be found in S1–S5 Tables.

After the <*BMD*_*i*,*post*−*Mission*_> for each group of astronauts was calculated, virtual diagnosis of osteopenia or osteoporosis was assigned based on the T-score by following WHO criteria. According to the international reference standard [22], osteoporosis is diagnosed when the T-score < 2.5, and osteopenia when 2.5 < T-score < -1. When T-score > -1, the T-score is considered normal.

## Results

The values obtained from the non-linear regression are summarized in Table 2. The predictive model for the BMD loss in the femoral neck of the astronauts during a spaceflight of length (*BMD*%(*t*)), based on the values listed in Table 2, is the following:

$$BMD\%(t)=(-69\%)e^{-0.0006371t}+69\%$$

**Fig 1:**
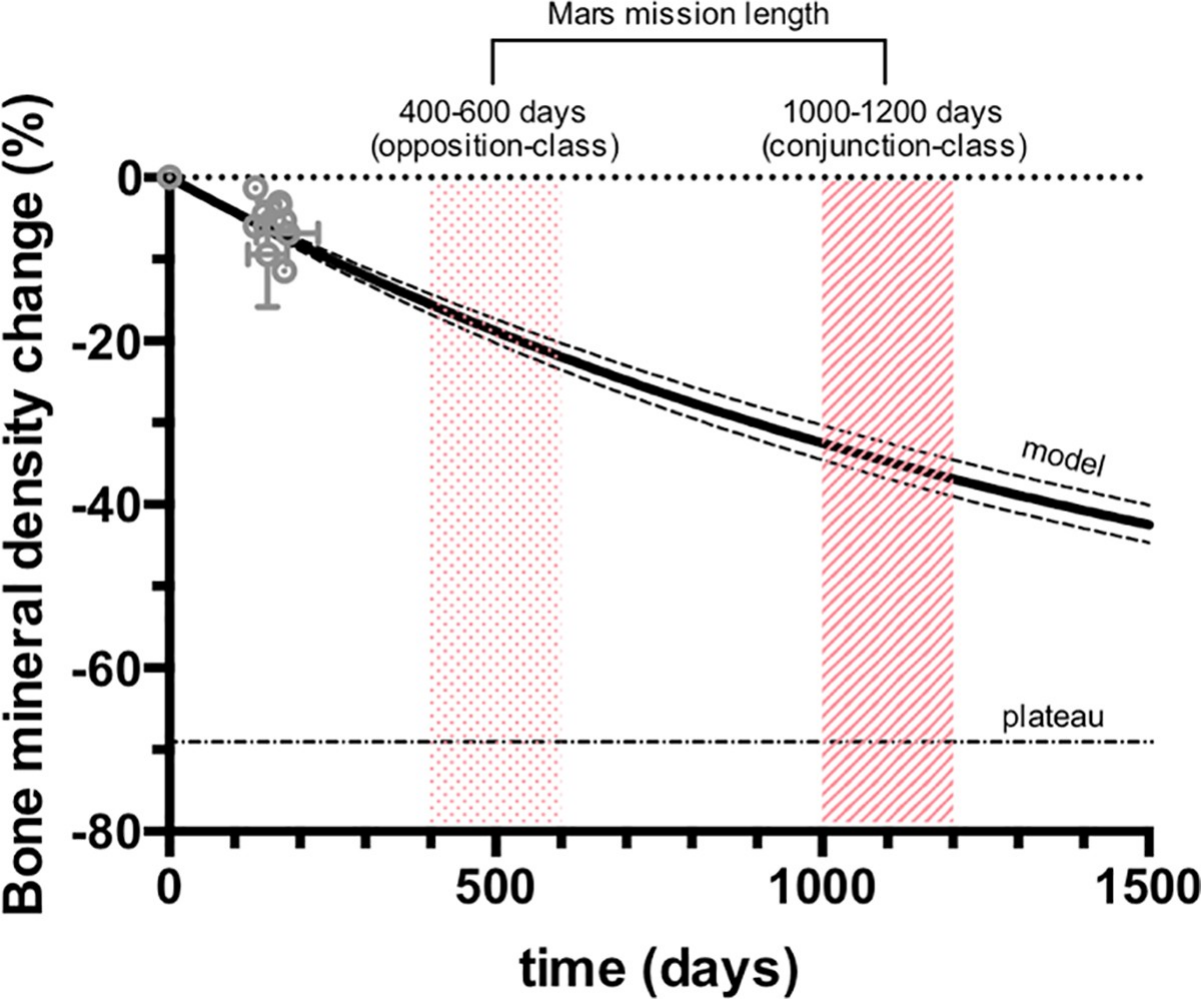
Bone mineral density change (%) at the femoral neck of astronauts *versus* duration of spaceflight. Grey dots represent experimental data obtained in previous missions as measured by dual-energy x-ray absorptiometry (DXA). Two different potential human missions to Mars are highlighted: (i) opposition-class, with a duration of 400–600 days (area with red dots) and (ii) conjunction-class, with a duration of 1000–1200 days (area with red lines). The predictive model is represented by the solid, black line, with the 95% confidence interval limits plotted in dashed, black lines, and the plateau by the dot-dashed, black line. A comparison with the (unphysical) linear model can be found in S1–S3 Figs.

Previous studies have calculated the duration of different strong candidate human missions to Mars [26,27]. Opposition class trajectory-based missions (400–600 days) also known as “short stay”, are technically and technologically demanding (*i*.*e*., they require high delta-velocity maneuvers and consequently require much higher propellant mass and would be challenging to perform with present propulsion technologies). Conjunction-class trajectories (1000–1200 days), also called “long stay” missions, minimize energy requirements and are less difficult to execute. By using our model, we predicted the BMD loss for these potential missions to Mars. In Fig 1, we plotted the previous function *versus* time in order to show its predictions for the BMD loss in long-duration spaceflights.

**Table 2:** Values of the parameters obtained for the mathematical model for the bone mineral density loss for long duration spaceflights.

**Parameter**	**Value**
Decay rate, ***λ***	6.371∙10^−4^ (a.u.)
Half life, *t*_1⁄2_	1088 days
Time constant, ***τ***	1570 days

Based on these results, we obtained T-scores or each gender/ethnicity/age group and summarized them in Table 3. A T-score is currently given in the clinic to compare the BMD of a patient to the BMD of a healthy young adult. Differences are measured in units named standard deviations (SDs). The more standard deviations below 0, the lower the BMD of the patient, and therefore, the higher the risk of fracture. A T-score between +1 and −1 is considered normal (healthy). A T-score between −1 and −2.5 indicates osteopenia. A T-score < −2.5 indicates osteoporosis.

**Table 3:** Estimated T-scores for each sex/ethnicity/age group. The color code is defined as by the international reference standard, osteoporosis is colored in red (T-score < -2.5), and osteopenia in orange (-2.5 < T-score < -1). Normal T-score is colored in green (T-score > -1). NASA´s non-permissible outcome is T-score < -2, highlighted by bold numbers in the table.

Mission time (days)	Mars mission trajectory	Astronaut age (yr)		Male			Female	
			Non-Hispanic white	Non-Hispanic black	Mexican American	Non-Hispanic white	Non-Hispanic black	Mexican American
400	Opposition-class (min)	30–39	-0.91	-0.08	-0.67	-1.35	-0.73	-1.05
		40–49	-1.25	-0.58	-1.03	-1.58	-0.72	-1.18
600	Opposition-class (max)	30–39	-1.38	-0.62	-1.16	-1.79	-1.22	-1.52
		40–49	-1.70	-1.08	-1.49	**-2.01**	-1.20	-1.64
1000	Conjunction-class (min)	30–39	**-2.20**	-1.49	-1.96	**-2.50**	**-2.01**	**-2.26**
		40–49	**-2.43**	-1.88	**-2.25**	**-2.69**	-1.99	**-2.38**
1200	Conjunction-class (max)	30–39	**-2.48**	-1.86	**-2.29**	**-2.80**	**-2.34**	**-2.58**
		40–49	**-2.73**	**-2.23**	**-2.57**	**-2.93**	**-2.33**	**-2.68**

## Discussion

We estimated fracture risk in astronauts in a potential mission to Mars as the output of the predictive model. We predict astronauts will lose 32.4–36.8% of their BMD in their femoral neck during the longer conjunction-class mission, which is the most likely duration of a human mission to Mars. By using mean values of BMD in the femoral neck, we predicted astronauts will return from such a mission with T-scores ranging from -1.49 to -2.93, depending on their pre-flight BMD. Thus, a subset of the astronauts is predicted to meet the diagnostic criteria for osteoporosis or osteopenia in a human mission to Mars. NASA´s medical standards for crewmember’s health establish a non-permissible T-score limit of -2. This means that to keep the skeletal health of crewmembers after a spaceflight, the T-score should be maintained above the non-permissible outcome limit. Yet, for the majority of the cases included in this analysis, bone loss exceeds the non-permissible limit (T-score < -2). Previous studies using linear models to estimate the risk of fragility fractures in crewmembers showed low risk on missions <1 year in duration [28]. This study predicts that significant risks of fragility fractures in crewmembers arise from the long duration of a future mission to Mars.

Even if it is more challenging from a technological point of view, an opposition class mission to Mars seems to be much safer with respect to risk for bone fracture (15.6–22.0% BMD loss in the femoral neck). Predicted T-scores range from -0.08 to -2.01, depending on sex, ethnicity, age and total duration of the mission. This means that an opposition class mission to Mars will present an acceptable risk of fracture (T-score > -2) for the majority of the astronauts. While some crew are likely to develop osteopenia, none are predicted to develop osteoporosis.

Consistent with terrestrial values from the NHANES reference database, non-Hispanic, 30–39 year old black males would have the lowest risk of bone fracture in any of the missions, while non-Hispanic, 40–49 years old, white females would have the highest risk for bone fracture amongst the different groups analyzed.

The mathematical model presented is the first to our knowledge that does not assume a linear decrease in the BMD of astronauts [28–30]. Although a linear decrease is widely used in the literature, it will eventually predict a negative BMDs in long-duration spaceflights, which lacks any physical meaning. Our model follows the terrestrial evidence that suggests that the BMD loss will eventually plateau [18] in long duration spaceflights. This approach allowed us to model the BMD loss in human interplanetary missions, providing a valuable tool for predicting fracture risk in astronauts for a mission to Mars.

Our model presents a simple and powerful mathematical tool enabling a quick estimation of the BMD loss and T-score for any astronaut in a long-duration spaceflight. Yet, some limitations arise. For example, our model does not take into account possible use of exercise, pharmaceutical (*e*.*g*., bisphosphonate), or dietary countermeasures that mitigate bone loss. The ability of countermeasure strategies to mitigate BMD loss of astronauts in missions exceeding 1 year in duration has not been tested. In this respect, more studies on the effects of countermeasures known to be effective in missions < 1 year, such the exercise with Advanced Resistive Exercise device (ARED)* are necessary to improve the predictive power of any model, including the one presented here [31]. The data used for formulating our model was obtained from spaceflights prior implementation of ARED in 2009. An additional limitation of our model is that the person-to-person variability of BMD loss observed in the available data [19,32,33] is large (see Fig 1). Further, the potential negative impact of prolonged exposure to deep space radiation on BMD is not taken into account, as all of the data used to generate the present model were obtained from astronauts in low earth orbit where some radioprotection is conferred by the magnetosphere. The limited spaceflight data is a limiting factor for our non-linear and traditional linear models. However, our non-linear model provides the most accurate and realistic prediction of the BMD loss in long-duration space flights to date. Once more data is obtained for the impact of deep space and long-duration spaceflight on BMD loss in humans, our model can be updated accordingly.

Our model offers the possibility of giving individual predictions for the T-score at any time point of the mission. In this study, the “pre-flight” BMDs input to obtain the T-scores were obtained from the NHANES reference database, while for an actual mission to Mars, individual pre-launch BMD of each astronaut would be need to supply data for input into the model. It is important to note that during missions, the risk of fracture is minimal as sudden mechanical overloads are very unlikely to happen in a microgravity environment. However, for astronauts landing on the surface of Mars, the risk of fracture increases, as falls or crushes would be more likely to happen in Mars’ fractional gravity (3.71 m/s^2^ *versus* 9.81 m/s^2^ on Earth), especially given the period of disuse during transit to Mars. The same logic applies to for astronauts after return to Earth, when the predicted BMD losses are presumably maximal.

Bone density is an important parameter that contributes to bone strength, but it would be both interesting and important to include other parameters that contribute to so-called “bone quality” in future studies (such as microarchitecture and material properties) to gain a more accurate estimate of fracture probability [23]. Unfortunately, due to the complexity of bone tissue, these contributors to “bone quality” are difficult to rigorously define and measure. There is evidence that trabecular microarchitecture and bone geometry affect the bone strength [34]. In this regard, magnetic resonance imaging (MRI) and high-resolution quantitative computed tomography (QCT) measurements of weight-bearing bones in crewmembers are encouraged to determine how spaceflights induce microcrack accumulation and/or resorption cavities over time. This could potentially help to fully capture and define how bone quality changes over time during long duration spaceflights. For the moment, our model can be used to predict T-scores from DXA scans and BMD values, which are typically available for crewmembers. Future MRI and QCT studies should provide more valuable data to inspiring more precise models. It is noteworthy to mention that this study can be also extended to other load-bearing bones apart from the femoral neck. Sibonga *et al*. demonstrated that spaceflight induced fracture risk increases not only in the femoral neck but in general [28]. In addition, we hope this non-linear model will also inspire future applications of a similar model to predict the bone mineral density loss of terrestrial patients under bed rest.

In conclusion, we have presented a mathematical model for BMD loss in crewmembers that can be used to predict T-scores during long-duration spaceflights. This model helps us understand the potential fracture risk for a potential mission to Mars, which is of paramount importance for NASA and other space agencies. T-scores < -2, NASA’s non-permissible outcome, were predicted for the 79% of the cases analyzed regarding a conjunction-class trajectory (the strongest candidate today for a human mission to the red planet). A bone fracture in an astronaut during a mission to Mars would endanger the mission goals and also could provoke medical complications (microgravity’s effect on bone cell performance could impair bone healing or induce sepsis or thromboembolic blood clots) that may result in morbidity or mortality. This model will help in designing mitigation strategies (use of ARED and adequate nutrition) for future human missions to Mars.

## Statistical analysis

Percent loss in bone mineral density at fermoral neck in astronauts (N = 69) after 400, 600, 1000 and 1200 day-spaceflights were statistically analyzed via unpaired t tests between all groups, whereby a two-tailed p value of p < 0.0001 was observed in all cases.

## Supporting information

S1 Fig Bone mineral density change at the femoral neck of astronauts predicted by our non-linear model and by the linear model versus length of spaceflight (0–10000 days). (PDF)

S2 Fig Bone mineral density change at the femoral neck of astronauts predicted by our non-linear model and by the linear model versus length of spaceflight (0–300 days). (PDF)

S3 Fig Bone mineral density change at the femoral neck of astronauts predicted by our non-linear model and by the linear model versus length of spaceflight (0–1500 days). (PDF)

S1 Table Predicted BMD loss in the femoral neck of crewmembers of different ages and ethnicities in an opposition-class mission to Mars with a total duration of 400 days. (PDF)

S2 Table Predicted BMD loss in the femoral neck of crewmembers of different ages and ethnicities in an opposition-class mission to Mars with a total duration of 600 days. (PDF)

S3 Table Predicted BMD loss in the femoral neck of crewmembers of different ages and ethnicities in a conjunction-class mission to Mars with a total duration of 1000 days. (PDF)

S4 Table Predicted BMD loss in the femoral neck of crewmembers of different ages and ethnicities in a conjunction-class mission to Mars with a total duration of 1200 days. (PDF)

S5 Table Predictions of percent loss in bone mineral density at fermoral neck in astronauts using data from astronauts (N = 69) after 132- to 228-day spaceflights. (PDF)
