# Supplementary material for: A human mission to Mars: Predicting the bone mineral density loss of astronauts
Source: PLoS One. 2020 Jan 22;15(1):e0226434. doi: 10.1371/journal.pone.0226434 (PMC6975633; doi:10.1371/journal.pone.0226434)
Supplement: S4 Table — (PDF) [file pone.0226434.s007.pdf]

Table S4. Predicted BMD loss in the femoral neck of crewmembers of different ages and ethnicities in a conjunction-class mission to Mars with a total duration of 1200 days.

	Male						Female					
Astronauts age (year)	Non-Hispanic white		Non-Hispanic black		Mexican American		Non-Hispanic white		Non-Hispanic black		Mexican American	
	Before mission	After mission	Before mission	After mission	Before mission	After mission	Before mission	After mission	Before mission	After mission	Before mission	After mission
30-39	0.887±0.134	0.561	1.005±0.158	0.635	0.922±0.127	0.583	0.825±0.120	0.521±	0.913±0.130	0.577	0.867±0.125	0.548
40-49	0.839±0.124	0.530	0.935±0.145	0.591	0.870±0.121	0.550	0.791±0.125	0.500±0.094	0.915±0.153	0.578	0.848±0.127	0.536
