# Supplementary material for: A human mission to Mars: Predicting the bone mineral density loss of astronauts
Source: PLoS One. 2020 Jan 22;15(1):e0226434. doi: 10.1371/journal.pone.0226434 (PMC6975633; doi:10.1371/journal.pone.0226434)
Supplement: S5 Table — (PDF) [file pone.0226434.s008.pdf]

Table S5. Predictions of percent loss in bone mineral density at fermoral neck in astronauts using data from astronauts (N=69) after 132- to 228-day spaceflights.

Mission time (days)	Mars mission (trajectory)	BMD loss predicition (%)
**400**	Opposition-class (min)	15.6±1.3
**600**	Opposition-class (max)	22.0±1.6
**1000**	Conjunction-class (min)	32.4±2.1
**1200**	Conjunction-class (max)	36.8±2.2
