# Supplementary material for: A human mission to Mars: Predicting the bone mineral density loss of astronauts
Source: PLoS One. 2020 Jan 22;15(1):e0226434. doi: 10.1371/journal.pone.0226434 (PMC6975633; doi:10.1371/journal.pone.0226434)
Supplement: S2 Fig — (PDF) [file pone.0226434.s002.pdf]

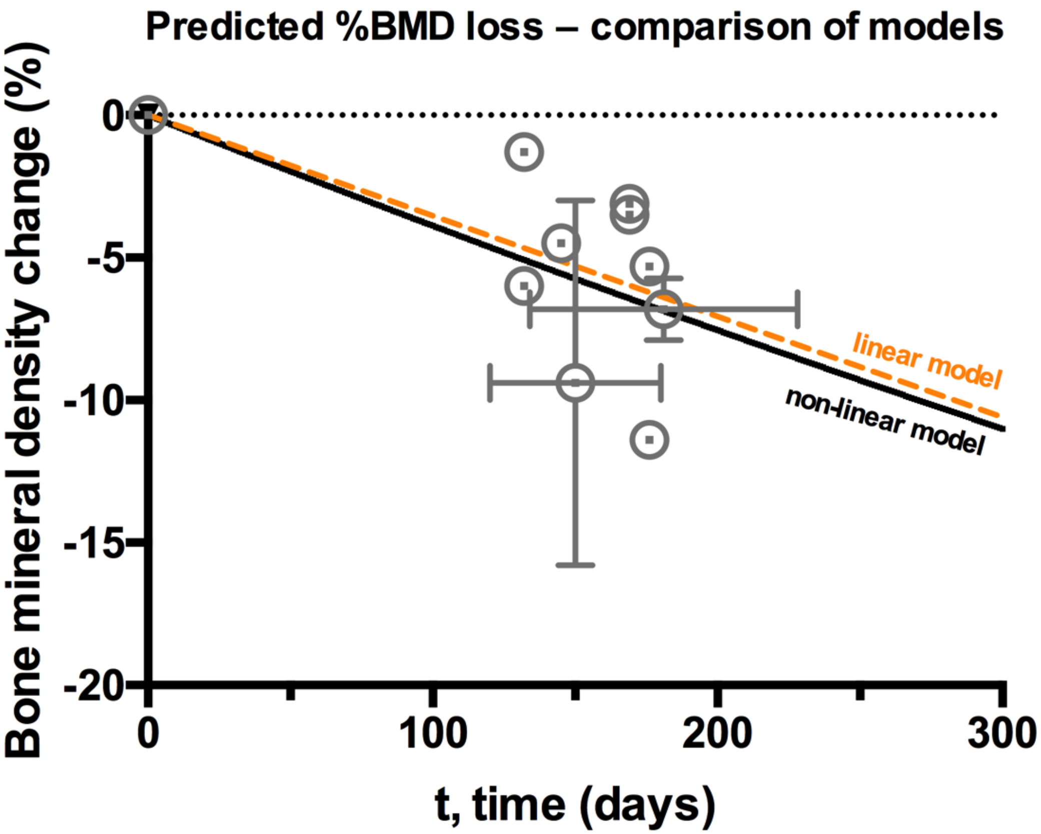

Figure S2. Bone mineral density change (%) at the femoral neck of astronauts predicted by our non-linear model (in solid, black line) and by the linear model (in dashed, orange line) *versus* length of spaceflight. Grey dots represent experimental data obtained in previous missions as measured by dual-energy x-ray absorptiometry (DXA).
